# Attention-Deficit/Hyperactivity Disorder and Bullying in School Settings: A Narrative Review of Implications for Prevention and Intervention Strategies

**DOI:** 10.3390/children13081047

**Published:** 2026-08-06

**Authors:** Felipe Montalva-Valenzuela, Eduardo Guzmán-Muñoz, Antonio Castillo-Paredes, Iván Molina-Márquez, Claudio Farias-Valenzuela, Rodrigo Yáñez-Sepúlveda, Guillermo Cortés-Roco, Álvaro Farfán-Díaz, Exal Garcia-Carrillo

**Affiliations:** 1 Escuela de Entrenador en Actividad Física y Deporte, Facultad de Ciencias Humanas, Universidad Bernardo O’Higgins, Santiago 8370993, Chile; felipe.montalva@ubo.cl; 2 Escuela de Fonoaudiología, Facultad de Ciencias de la Salud, Universidad Autónoma de Chile, Talca 3460000, Chile; 3 Grupo AFySE, Investigación en Actividad Física y Salud Escolar, Escuela de Pedagogía en Educación Física, Facultad de Educación, Universidad de Las Américas, Santiago 8370040, Chile; acastillop85@gmail.com; 4 Pedagogía en Educación Física, Facultad de Educación, Universidad Adventista de Chile, Chillán 3780000, Chile; ivanmolina@unach.cl; 5 Programa Doctorado en Ciencias de la Actividad Física, Universidad Católica del Maule, Talca 3460000, Chile; 6 Escuela de Ciencias de la Actividad Física, el Deporte y la Salud, Universidad de Santiago de Chile (USACH), Santiago 9170022, Chile; claudio.farias.v@usach.cl; 7 Facultad de Educación y Humanidades, Escuela de Ciencias del Deporte, Universidad Andres Bello, Viña del Mar 2200055, Chile; rodrigo.yanez.s@unab.cl; 8 School of Medicine, Universidad Espíritu Santo, Samborondón 092301, Ecuador; 9 Facultad de Ciencias de la Vida, Magíster en Evaluación y Planificación del Entrenamiento Deportivo, Universidad Viña del Mar, Viña del Mar 2520000, Chile; guillermo.cortes@uvm.cl; 10 Departamento de Nutrición y Kinesiología, Carrera de Nutrición, Facultad de Ciencias de la Salud, Universidad de Tarapacá, Arica 1000000, Chile; alvarofarfandiaz@gmail.com; 11 Department of Physical Activity Sciences, Faculty of Education Sciences, Universidad Católica del Maule, Talca 3480112, Chile; exal.garcia@gmail.com; 12 Department of Physical Activity Sciences, Universidad de Los Lagos, Osorno 5290000, Chile

**Keywords:** attention-deficit/hyperactivity disorder, bullying, school bullying, cyberbullying, peer victimization

## Abstract

**Highlights:**

**What are the main findings?**

• Children and adolescents with ADHD show greater involvement in bullying, including victimization, perpetration, bully–victim profiles, and cyberbullying, with victimization being the most consistently reported outcome.
• Bullying involvement appears to reflect the interaction of ADHD-related characteristics with emotional, peer-, family-, and school-related factors rather than the isolated effect of core ADHD symptoms.

**What are the implications of the main findings?**

• Bullying assessment in young people with ADHD should consider multiple forms of involvement and the influence of individual, family, peer, and school contexts.
• Prevention and intervention strategies may benefit from strengthening protective factors across school, family, and peer environments rather than focusing exclusively on ADHD symptoms.

**Abstract:**

Background: Attention-deficit/hyperactivity disorder (ADHD) is associated with cognitive, behavioral, emotional, and social difficulties that may increase vulnerability to bullying during childhood and adolescence. Although previous studies have examined different aspects of this relationship, the available evidence remains limited and heterogeneous, particularly regarding associated factors, profiles of bullying involvement, and implications for prevention and intervention. Objective: To analyze the available evidence regarding bullying involvement in children and adolescents with ADHD, focusing on victimization, perpetration, bully–victim profiles, associated factors, psychological consequences, and implications for prevention and intervention. Methods: A narrative review was conducted using studies identified in PubMed, Scopus, and Web of Science between January and March 2026, with no publication date restrictions. The search was performed in three independent and consecutive stages and included observational studies evaluating the relationship between ADHD and bullying in children and adolescents. Narrative reviews, systematic reviews, meta-analyses, editorials, and studies conducted exclusively in adults were excluded. Results: Seventeen studies were included. The evidence consistently showed that children and adolescents with ADHD have greater involvement in bullying than their peers without ADHD. Victimization was the most consistently reported outcome, although increased perpetration, bully–victim profiles, and cyberbullying were also identified. Bullying involvement appeared to be influenced by multiple interacting factors, including ADHD symptoms, emotional dysregulation, impulsivity, comorbidities, peer relationships, family characteristics, and school-related factors. Victimization was consistently associated with increased anxiety and depressive symptoms, emotional difficulties, and poorer quality of life. Emerging evidence also suggests sex-specific patterns of bullying involvement and bidirectional longitudinal relationships between ADHD symptoms and bullying experiences. Conclusions: Children and adolescents with ADHD may have greater involvement in multiple forms of bullying; however, this relationship appears to be multifactorial rather than solely explained by core ADHD symptoms. Family, peer, and school environments may play important protective roles, highlighting the need for comprehensive prevention and intervention strategies that address both individual and contextual factors.

## 1. Introduction

Attention-deficit/hyperactivity disorder (ADHD) is one of the most common neurodevelopmental disorders in childhood [1] and is characterized by three core symptoms: inattention, hyperactivity, and impulsivity [2]. The prevalence of ADHD is higher in males than in females, with reported male-to-female ratios ranging from 3:1 to 16:1 [3], although this difference tends to be smaller in community-based samples, approaching 3:1 [4]. This discrepancy between clinical and community samples may be partly explained by the underrecognition and underdiagnosis of ADHD in females, who often present with less overt hyperactive and disruptive symptoms and are therefore less likely to be referred for clinical assessment [5]. This condition is associated with significant impairments in cognitive and behavioral functioning, particularly in executive functions, including inhibitory control, working memory, and planning, which may affect the personal, social, and even occupational development of affected individuals [2,6,7]. In this regard, impairments in executive functions have been consistently associated with poorer academic performance, particularly in tasks requiring sustained self-regulation and behavioral organization over time [8,9].

Compared with children without ADHD symptoms, those with this condition tend to report a lower quality of life [10], together with a higher likelihood of developing anxiety and depressive symptoms [11]. These difficulties are reflected not only at the individual level but also in social and school functioning, where poorer social skills with peers and less close, more conflictive relationships with teachers have been reported [12,13]. In this context, the impact of ADHD extends beyond academic performance, also affecting classroom behavior [14]. In addition, adapting to everyday demands may be challenging, as symptom expression can fluctuate from day to day [15].

The school environment represents a key setting in which these difficulties may be amplified, particularly through peer victimization. Bullying is defined as a form of interpersonal violence characterized by repeated aggressive behaviors occurring over time within a context of power imbalance between the perpetrator and the victim, and includes physical, verbal, and relational forms of aggression [16,17]. Over the past decade, this phenomenon has expanded into the digital environment under the term cyberbullying [18], which involves the use of technology to perpetrate persistent harassment with greater reach and public exposure. Both forms of victimization have been associated with significant adverse mental health outcomes, including depressive symptoms, suicidal ideation and behavior, school dropout, and poorer performance on standardized academic tests [19,20].

In this regard, children and adolescents with ADHD may represent a particularly vulnerable group to peer victimization due to characteristics such as impulsivity, difficulties in emotional regulation, and potential impairments in the interpretation of social cues. These characteristics may increase the risk of both being victims of bullying and engaging in perpetration within the school setting. Furthermore, the available evidence suggests that bullying is a multifactorial phenomenon influenced by the interaction of individual, social, and school-related factors [21]. Taken together, these findings suggest that children and adolescents with ADHD may have greater involvement in bullying than their peers without ADHD, although the magnitude of this association and the mechanisms underlying it remain insufficiently understood. Although previous studies have examined different aspects of this relationship, the available evidence remains limited and heterogeneous, particularly regarding factors associated with bullying involvement, profiles of bullying involvement, and their implications for prevention and intervention.

Therefore, the present narrative review aimed to analyze the available evidence on bullying in children and adolescents with ADHD by integrating findings related to victimization, perpetration, and mixed forms of involvement. This review sought to synthesize the main factors associated with this phenomenon, including cognitive, emotional, social, and school-related aspects that may contribute to its occurrence. Finally, the implications of these findings for prevention and intervention in educational settings were discussed to contribute to a better understanding of this issue.

## 2. Materials and Methods

A narrative review was conducted because the objective was to provide an integrative synthesis of heterogeneous evidence rather than estimate pooled effect sizes (adaptation of the PRISMA Checklist available in Supplementary Table S1). Accordingly, a literature search was conducted between January and March 2026 in the PubMed, Web of Science, and Scopus databases, with no publication date restrictions, to identify studies examining the relationship between ADHD and bullying in children and adolescents. To maximize the identification of relevant studies, the search was performed in three independent and consecutive stages. In the first stage, general terms related to ADHD and bullying were used. Subsequently, a second search was conducted, incorporating more specific terms related to victimization, perpetration, and other forms of involvement in peer bullying. Finally, a manual review of the reference lists of the selected studies was performed to identify additional publications relevant to the topic of this review.

The search strategy included combinations of terms related to ADHD and bullying. The keywords used included “ADHD”, “attention deficit hyperactivity disorder”, “attention deficit”, “bullying”, “peer victimization”, “peer aggression”, “victimization”, “perpetration”, “cyberbullying”, “school bullying”, “children”, “adolescent”, and “youth”. These terms were combined using Boolean operators (“AND” and “OR”), adapting the search strategy according to the characteristics of each database.

Observational, cross-sectional, longitudinal, cohort, and case–control studies evaluating the relationship between ADHD and bullying in children and adolescents were considered eligible. Studies including participants with a clinical diagnosis of ADHD, as well as studies assessing ADHD symptoms using validated instruments, were included, since a substantial proportion of the epidemiological literature adopts a dimensional approach to examine the association between symptoms of inattention and hyperactivity/impulsivity and involvement in bullying. Studies examining victimization, perpetration, mixed forms of involvement, or cognitive, emotional, behavioral, family, or school-related factors associated with these experiences were also included.

Narrative reviews, systematic reviews, meta-analyses, editorials, letters to the editor, study protocols, and studies that did not report specific outcomes related to bullying, victimization, or peer perpetration were excluded. Studies conducted exclusively in adult populations were also excluded.

Only articles published in English were included, as English is the predominant language of publication in the databases searched (PubMed, Scopus, and Web of Science) and provides broad coverage of the international scientific literature. Titles and abstracts were initially screened to determine their relevance to the review topic. Subsequently, the selected studies underwent full-text assessment to verify compliance with the eligibility criteria described above and confirm their final inclusion.

The collected evidence was analyzed narratively, organizing the findings according to the main themes identified: involvement in bullying, associated factors, psychological and social consequences, special considerations, and implications for prevention and intervention in school settings. Given the narrative nature of the review, no formal risk-of-bias assessment was performed.

## 3. Results

### 3.1. Involvement in Bullying Among Children and Adolescents with ADHD

**Figure 1:**
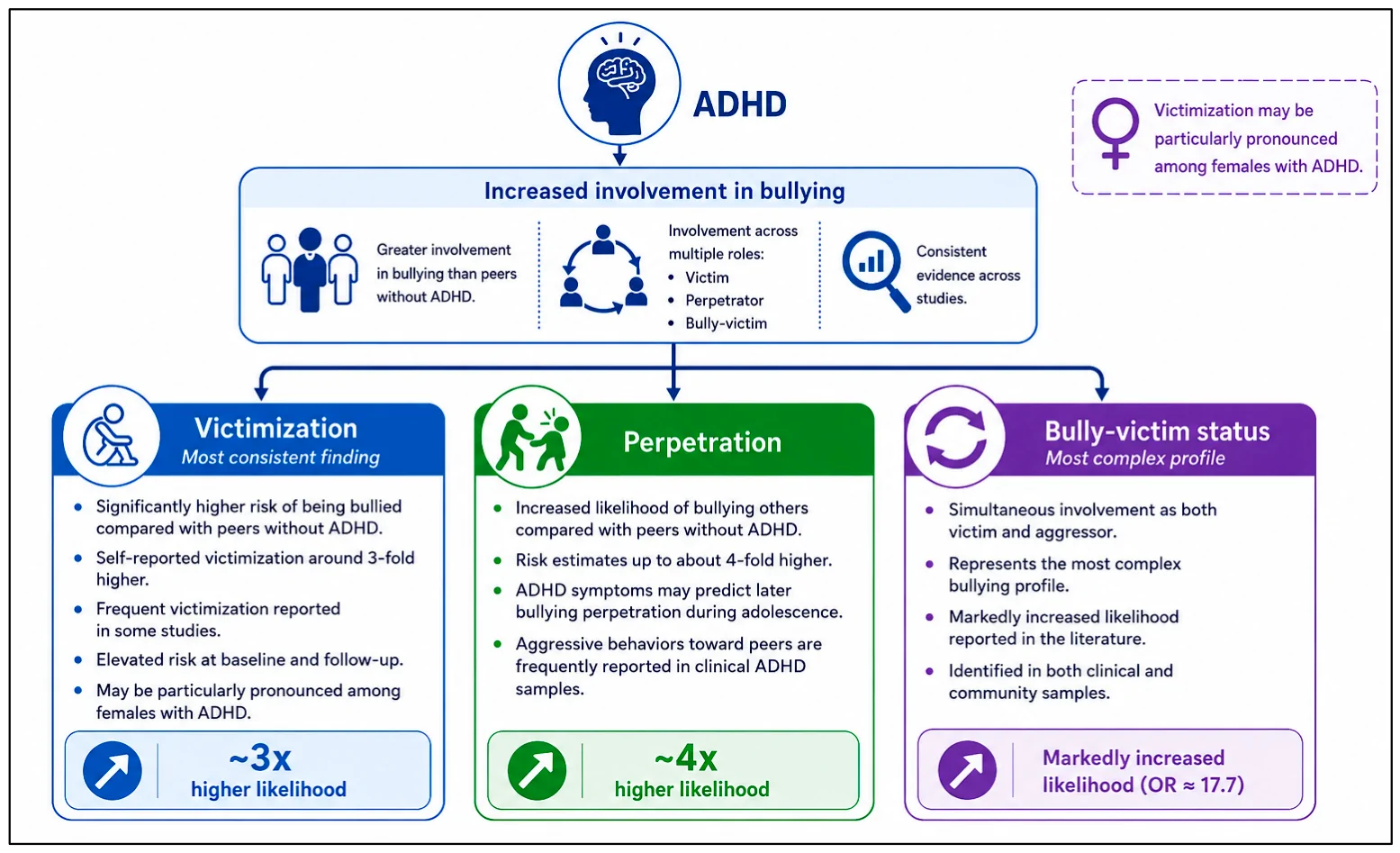
Summary of the main forms of bullying involvement reported in children and adolescents with ADHD. The available evidence consistently indicates an increased participation in victimization, bullying perpetration, and bully–victim profiles compared with peers without ADHD. (OR = Odds ratio).

The available evidence consistently shows that children and adolescents with ADHD are more likely to be involved in bullying than their peers without the disorder [22,23,24,25,26,27,28,29,30,31,32,33,34,35,36]. This increased involvement is not limited to victimization but also includes a greater likelihood of engaging in bullying perpetration and, in some cases, simultaneously participating as both victims and perpetrators [23,27,28,30,32,36] (Figure 1).

Victimization was the most consistent finding across the studies reviewed. Several studies reported that children and adolescents with ADHD were at a significantly greater risk of experiencing bullying than those without the disorder [22,24,26,29,30,31,32,33,34,35,36]. In a study conducted in preadolescents, Voltas et al. [24] found that ADHD was associated with an almost threefold higher likelihood of self-reported victimization compared with controls. Similarly, Holmberg and Hjern [30] reported that children with ADHD were significantly more likely to experience frequent victimization (odds ratio (OR) = 10.8). Likewise, Orengül et al. [22] found that schoolchildren with ADHD presented significantly higher levels of victimization than their peers without the disorder, both at baseline and after one year of follow-up. Along the same line, Accardo et al. [31] reported high rates of victimization among young people with ADHD, particularly among females, reinforcing the consistency of this association across different settings and age groups.

In addition to victimization, several studies reported a greater involvement of young people with ADHD as perpetrators of bullying [23,25,27,28,29,30,32,33,36]. Holmberg and Hjern [30] found that children with ADHD were approximately four times more likely to engage in bullying behaviors toward their peers. Using a longitudinal design, Murray et al. [25] further reported that ADHD symptoms predicted subsequent increases in bullying perpetration during adolescence, suggesting that certain characteristics associated with the disorder may contribute to the development of these behaviors over time. Likewise, studies conducted in clinical samples of children and adolescents with ADHD reported a higher frequency of aggressive behaviors toward peers than that typically observed in the general population [23,27,28].

A particularly relevant finding was the presence of a subgroup of young people with ADHD who simultaneously participated as both victims and perpetrators, commonly referred to as bully–victims [23,27,28,30,32,36]. The available evidence suggests that this profile represents one of the most complex forms of involvement within this population. Fogler et al. [32] observed that individuals with a history of ADHD were markedly more likely to belong to this category (odds ratio (OR) = 17.71), whereas Chou et al. [23] identified the simultaneous presence of both roles among adolescents with a clinical diagnosis of ADHD. Taken together, these findings suggest that ADHD is associated with a greater vulnerability to involvement in different forms of bullying, whether as a victim, perpetrator, or through mixed patterns of participation.

### 3.2. Factors Associated with Bullying Involvement in Children and Adolescents with ADHD

**Figure 2:**
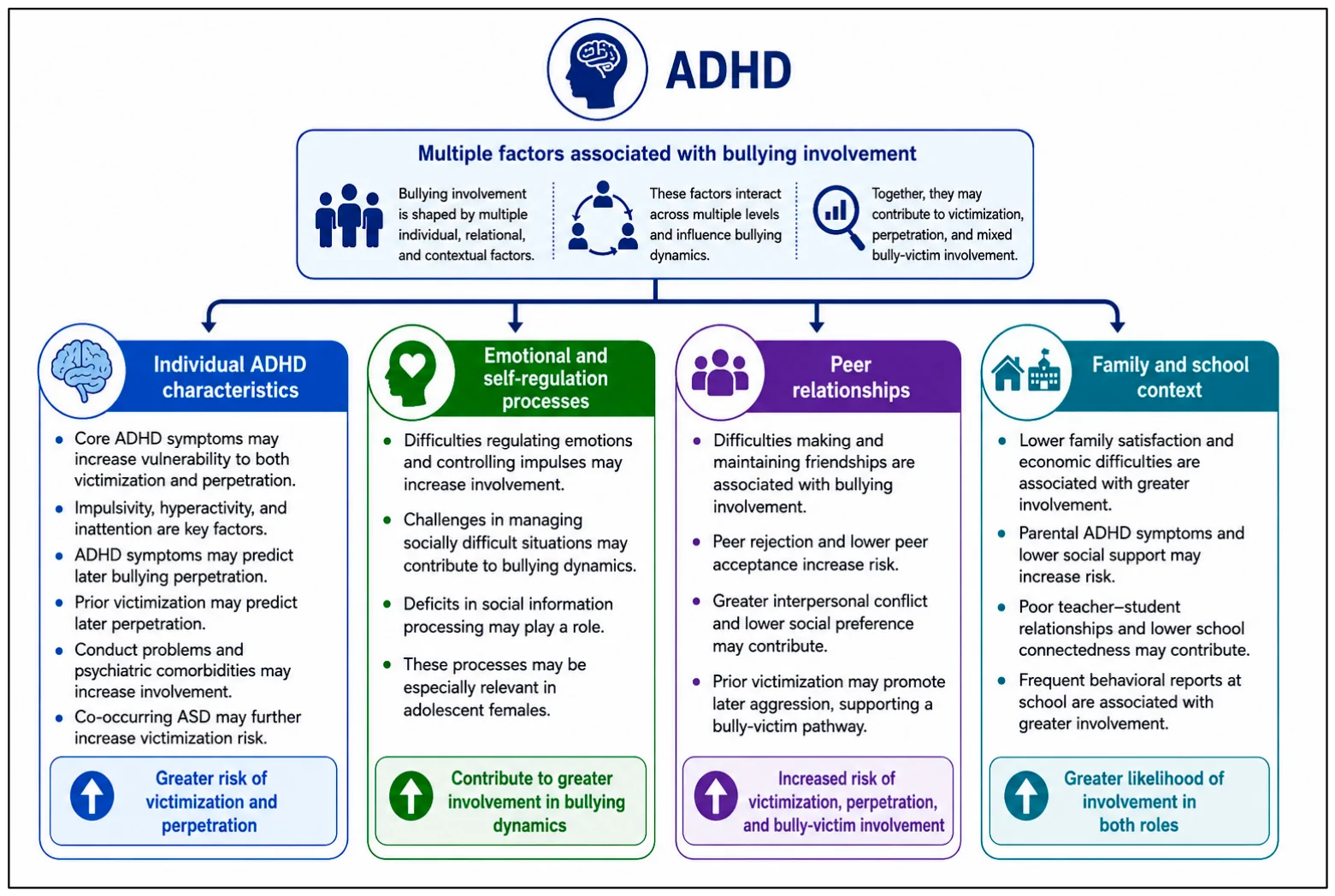
Summary of the main factors associated with bullying involvement in children and adolescents with ADHD. The available evidence suggests that bullying involvement is influenced by the interaction of individual, family, and contextual factors.

Bullying involvement among children and adolescents with ADHD appears to be influenced by multiple individual, family, and contextual factors (Figure 2). Several studies suggest that the core characteristics of the disorder, particularly impulsivity, hyperactivity, and attentional difficulties, may increase vulnerability to both victimization and bullying perpetration [24,25,28,29,34,36]. In this regard, Ahmed et al. [29] found that students involved in bullying behaviors exhibited significantly higher scores for conduct problems, inattentive symptoms, and hyperactivity than those not involved in these dynamics. Similarly, Murray et al. [25] reported that childhood ADHD symptoms predicted greater subsequent involvement in bullying perpetration. In contrast, Bong et al. [28] found that impulsivity was significantly associated with bullying behaviors, identifying it as one of the main factors related to perpetration. In addition, they found that previous victimization was the strongest predictor of these behaviors, suggesting that some children may transition from the role of victim to that of perpetrator. Furthermore, the presence of comorbidities appears to represent an additional risk factor. Chou et al. [23] found that adolescents with ADHD and autism spectrum disorder (ASD) were more likely to experience bullying victimization than those without this comorbidity. Similarly, Voltas et al. [24] reported a higher prevalence of ASD among participants with ADHD who had experienced bullying victimization, suggesting that the social difficulties associated with this condition may increase vulnerability to bullying. More broadly, Fogler et al. [32] found that the coexistence of ADHD with other psychiatric disorders was also associated with greater involvement in bullying. Taken together, these findings suggest that the social, emotional, and behavioral difficulties associated with certain comorbidities may increase the risk of involvement in bullying.

Beyond the core symptoms of the disorder, several authors have highlighted the importance of emotional and self-regulatory processes. Recent studies suggest that difficulties in regulating emotional responses, controlling impulses, and managing socially challenging situations may contribute to both victimization and bullying perpetration [26,31,36]. Kim et al. [36] discussed that factors related to social information processing, emotional regulation, and peer relationships may help explain the greater involvement in bullying observed among adolescents with ADHD, particularly females.

Peer relationships represent another factor consistently associated with bullying involvement. Several studies have identified that difficulties in establishing and maintaining friendships, peer rejection, and lower peer acceptance are significantly associated with an increased risk of involvement in bullying [23,27,33,36]. Bustinza et al. [27] found that difficulties in making or maintaining friendships were strongly associated with victimization, whereas Mastrokoukou et al. [33] reported that lower social preference and greater conflict in interpersonal relationships were associated with greater involvement in bullying. Furthermore, some findings suggest that previous victimization may be associated with subsequent aggressive behaviors, supporting the possibility of an interaction cycle that may help explain the presence of individuals who simultaneously participate as both victims and perpetrators [28,32].

Finally, several family and school-related factors also appear to play an important role. Lower family satisfaction, economic difficulties, parental ADHD symptoms, and lower levels of social support have been associated with a greater likelihood of both victimization and perpetration [23,27,28,35]. Likewise, school-related variables, including poor teacher–student relationships, lower school connectedness, and frequent reports of behavioral problems, have been associated with greater involvement in bullying [27,33,35]. Taken together, these findings suggest that bullying involvement among children and adolescents with ADHD reflects the interaction of multiple individual and contextual factors rather than the isolated presence of the core symptoms of the disorder.

### 3.3. Consequences of Bullying Involvement in Children and Adolescents with ADHD

**Figure 3:**
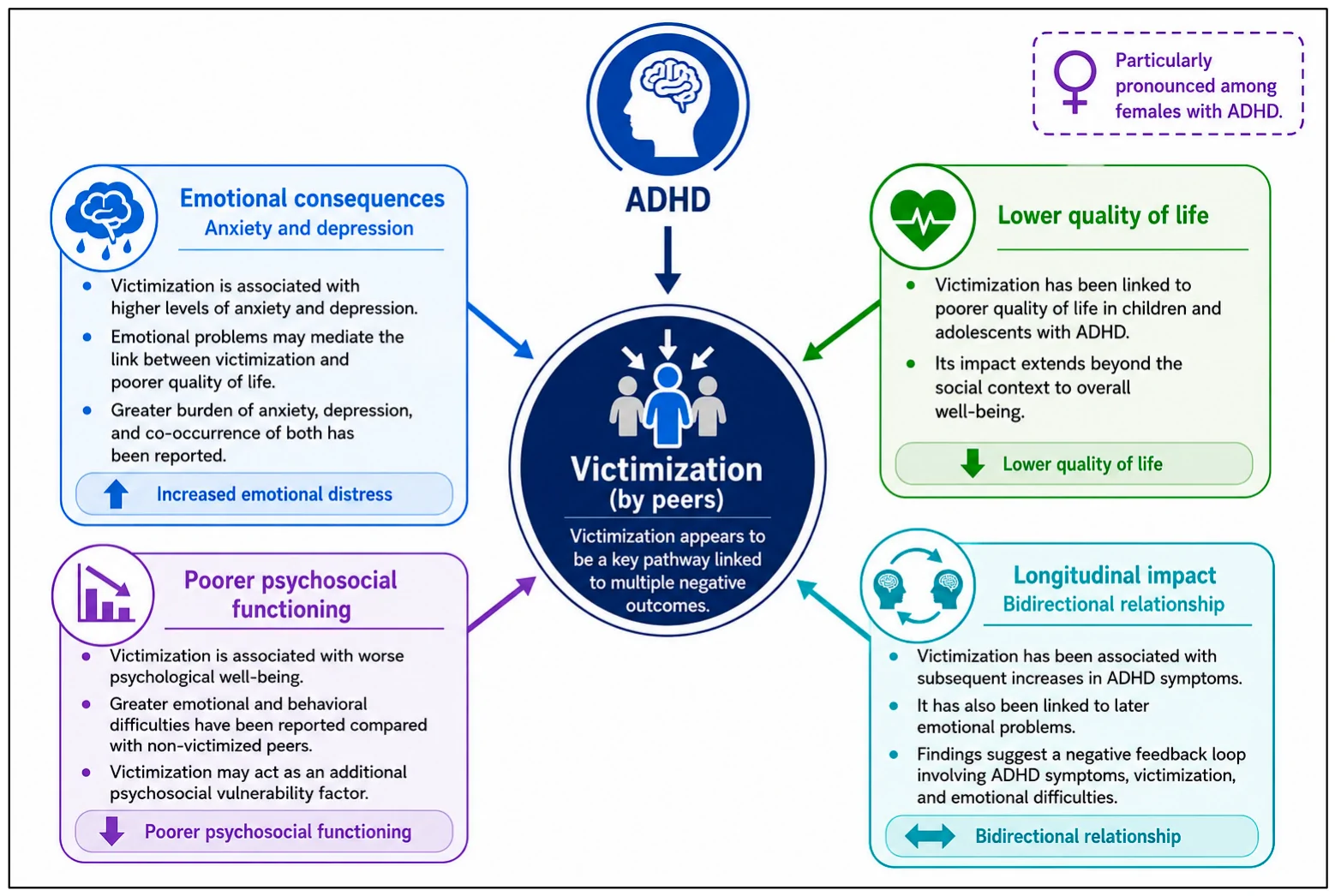
Summary of the main psychological and social consequences associated with bullying involvement in children and adolescents with ADHD. The available evidence suggests that peer victimization is associated with poorer mental health and quality of life in this population.

Bullying involvement among children and adolescents with ADHD has been associated with several psychological, social, and functional consequences (Figure 3). Although most of the included studies used cross-sectional designs, the available evidence suggests that peer victimization may be associated with greater emotional difficulties and poorer overall functioning in this population [22,24,26,31,34].

Among the most frequently reported consequences were internalizing problems, particularly symptoms of anxiety and depression. Orengül et al. [22] found that, among schoolchildren with ADHD, victimization was associated with greater internalizing problems and poorer quality of life. Similarly, Lin et al. [26] reported that the association between bullying victimization and poor quality of life in adolescents with ADHD was primarily mediated by emotional problems, including depressive and anxiety symptoms. These findings suggest that the impact of bullying extends beyond the immediate social experience, affecting both emotional well-being and overall perceived quality of life.

The available evidence also indicates that the association between bullying and psychological distress may be particularly relevant in certain subgroups. Accardo et al. [31] reported that bullying victimization among young people with ADHD was associated with higher rates of anxiety, depression, and the coexistence of both conditions, with a particularly high burden observed among females. Along the same line, Voltas et al. [24] found that schoolchildren with ADHD who reported bullying victimization presented greater emotional and behavioral difficulties than those without experiences of bullying, reinforcing the notion that victimization may act as an additional psychosocial vulnerability factor.

From a longitudinal perspective, some studies suggest that the relationship between ADHD, victimization, and emotional difficulties may be bidirectional. Stenseng et al. [34] observed that peer victimization was not only associated with greater inattentive symptoms over time but also with subsequent emotional problems. These findings suggest that bullying may contribute to maintaining or worsening attentional and emotional difficulties, creating a negative feedback cycle involving symptoms, peer relationships, and psychological well-being.

Taken together, these findings indicate that bullying involvement, particularly victimization, may have important implications for the mental health and quality of life of children and adolescents with ADHD. However, given the heterogeneity of the study designs and the predominance of cross-sectional studies, these findings should be interpreted with caution. Nevertheless, the available evidence highlights the importance of considering peer victimization as a relevant component of the clinical, educational, and psychosocial assessment of this population.

### 3.4. Emerging Evidence and Special Considerations Regarding Bullying in Young People with ADHD

**Figure 4:**
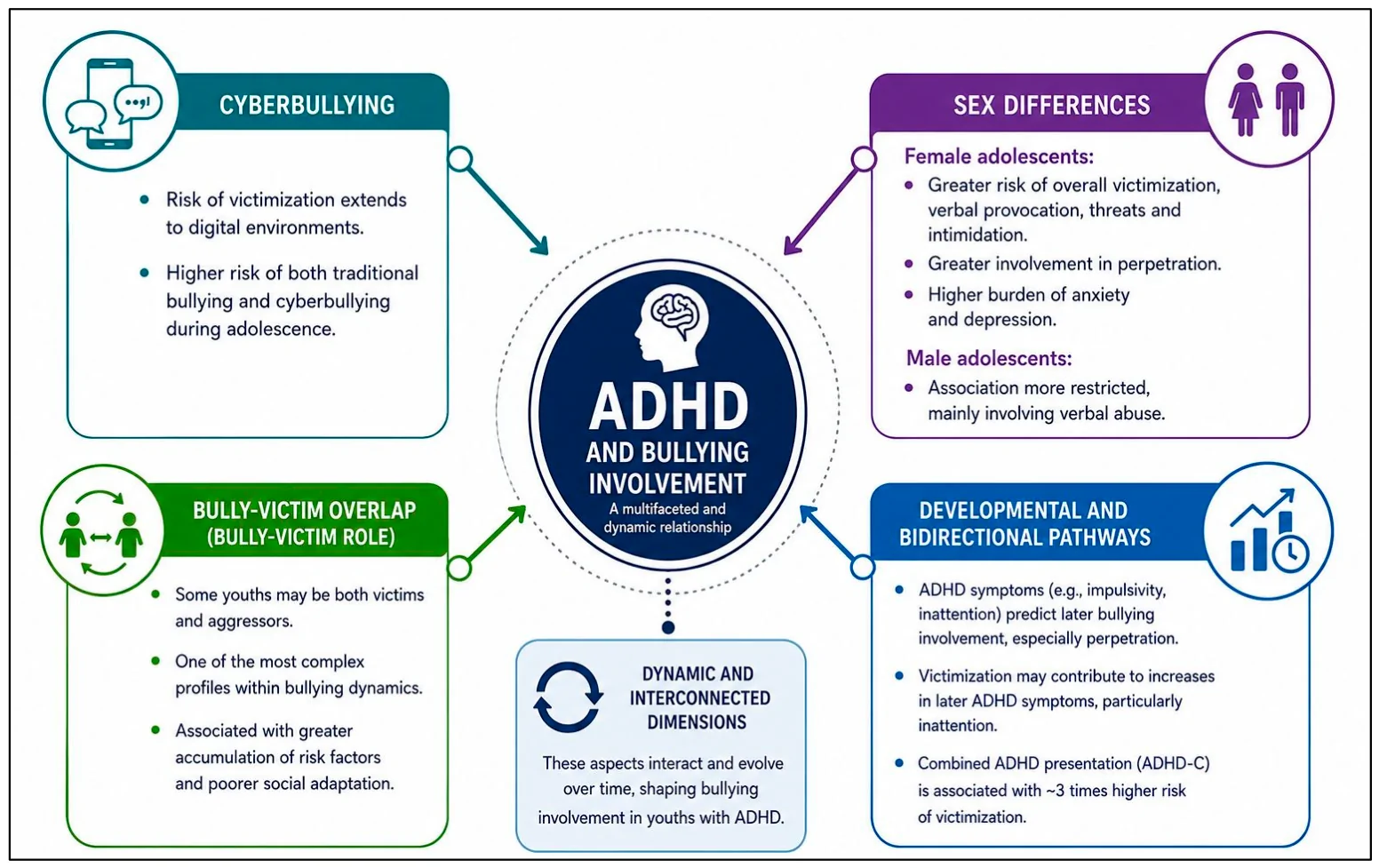
Summary of emerging evidence and special considerations regarding bullying in children and adolescents with ADHD. The available evidence highlights the relevance of cyberbullying, sex differences, bully–victim profiles, and longitudinal findings in understanding bullying involvement in this population.

In addition to the traditional forms of bullying, some studies have begun to explore specific manifestations of this phenomenon in children and adolescents with ADHD (Figure 4). Among these, cyberbullying has received increasing attention due to the growing use of digital social interactions. In this regard, Lin et al. [35] found that an ADHD diagnosis during childhood was associated with a greater risk of victimization through both traditional bullying and cyberbullying during adolescence. These findings suggest that the social vulnerabilities associated with ADHD may extend beyond the face-to-face school environment and persist in digital settings.

Another relevant aspect concerns sex differences. Accardo et al. [31] reported a greater burden of anxiety and depressive symptoms among young people with a history of bullying victimization, with particularly strong associations observed among females. Likewise, Kim et al. [36] identified a sex-specific pattern: among females, ADHD symptoms were associated with a greater likelihood of overall victimization, verbal provocation, threats and intimidation, as well as greater involvement in bullying perpetration. In contrast, among males, this association was more limited and was observed mainly for verbal abuse. These findings suggest that the relationship between ADHD and bullying may differ according to sex, with a possible greater overlap between victimization and perpetration among adolescent females.

Several studies have also highlighted the overlap between the roles of victim and perpetrator. This phenomenon, commonly referred to as the bully–victim profile, has been frequently described among young people with ADHD and appears to represent one of the most complex forms of involvement in bullying [23,27,28,30,32,36]. The available evidence suggests that some children and adolescents may alternate or combine both roles, possibly because of difficulties in emotional regulation, problems in peer relationships, and previous experiences of victimization. Identifying this profile is particularly relevant, as these individuals often present an accumulation of risk factors and greater difficulties in social adjustment.

Finally, the available longitudinal evidence suggests that the relationship between ADHD and bullying may persist throughout development and involve bidirectional influences. Murray et al. [25] found that ADHD symptoms predicted greater subsequent involvement in bullying perpetration, suggesting that difficulties related to impulsivity, behavioral regulation, and social skills may contribute to aggressive responses in conflict situations. In turn, Stenseng et al. [34] observed a reciprocal relationship between inattentive symptoms and bullying victimization. Specifically, higher levels of inattention predicted a greater risk of subsequent victimization, whereas experiences of victimization were associated with subsequent increases in inattentive symptoms. Likewise, Voltas et al. [24] reported that approximately one in three preadolescents with ADHD experienced bullying victimization, with the highest prevalence observed among those with the combined presentation of ADHD (ADHD-C) compared with the predominantly inattentive and predominantly hyperactive-impulsive presentations. Furthermore, after adjusting for several sociodemographic and clinical variables, ADHD-C was associated with an almost threefold higher risk of victimization, suggesting that the coexistence of inattention, hyperactivity, and impulsivity may increase vulnerability to bullying. Taken together, these findings reinforce the notion that the relationship between ADHD and bullying is dynamic and multifactorial, suggesting that specific symptoms of the disorder may increase the risk of involvement in bullying, whereas victimization itself may contribute to maintaining or exacerbating some of these manifestations over time.

## 4. Integrative Perspective on ADHD and Bullying

The findings of this narrative review suggest that the relationship between ADHD and bullying should not be understood as a direct consequence of the disorder itself. Bullying involvement appears to reflect the interaction between ADHD-related characteristics and multiple interpersonal and contextual factors [21]. Although victimization was the most consistently reported form of involvement [22,24,26,29,30,31,32,33,34,35,36], the evidence also identified increased perpetration [23,25,27,28,29,30,32,33,36] and a marked presence of bully–victim profiles [23,27,28,30,32,36]. These findings are consistent with evidence showing increased involvement in victimization, perpetration, and perpetration-victimization among children and adolescents with neurodevelopmental and psychiatric conditions compared with controls [37]. Thus, children and adolescents with ADHD may become involved in bullying through different and sometimes overlapping roles.

Several characteristics associated with ADHD may contribute to this vulnerability. Impulsivity has been associated with bullying perpetration [28], while inattentive symptoms, hyperactivity, and conduct problems were more frequent among students involved in bullying [29]. Difficulties in emotional regulation, impulse control, and social information processing may also contribute to victimization and perpetration [26,31,36]. Longitudinal evidence indicates that ADHD symptoms may predict subsequent bullying perpetration [25], whereas inattentive symptoms may predict subsequent victimization [34]. These characteristics occur alongside difficulties in peer relationships, including lower peer acceptance, problems establishing and maintaining friendships, and interpersonal conflict [23,27,33,36]. Together, these findings suggest that individual characteristics and peer experiences may jointly shape patterns of bullying involvement among young people with ADHD.

Victimization appears to occupy a particularly relevant position within this interaction. In addition to being the most consistently reported form of bullying involvement [22,24,26,29,30,31,32,33,34,35,36], previous victimization was identified as the strongest predictor of subsequent bullying behaviors in one clinical sample of children with ADHD [28]. This finding may help explain, at least partly, the frequent overlap between victim and perpetrator roles observed among young people with ADHD [23,27,28,30,32,36]. Rather than representing completely independent categories, victimization and perpetration may coexist or occur at different stages of development, contributing to the presence of bully–victim profiles. The longitudinal relationship between inattentive symptoms and peer victimization further supports the possibility of bidirectional interactions, in which inattentive symptoms may increase vulnerability to subsequent victimization, while victimization may contribute to later increases in inattentive symptoms [34].

The consequences associated with bullying involvement add further complexity to this relationship. Victimization among children and adolescents with ADHD has been associated with internalizing problems, anxiety and depressive symptoms, emotional and behavioral difficulties, and poorer quality of life [22,24,26,31,34]. Importantly, emotional problems, including depressive and anxiety symptoms, mediated the association between bullying victimization and poorer quality of life in adolescents with ADHD [26]. These findings are also consistent with broader evidence indicating that bullying involvement among young people with neurodevelopmental and psychiatric conditions is associated with greater internalizing and externalizing symptom severity [37]. These findings indicate that bullying experiences may represent an additional source of psychological vulnerability in young people who may already experience difficulties in emotional, behavioral, and social functioning. Consequently, bullying involvement should be considered as part of the broader psychosocial context of children and adolescents with ADHD rather than as an isolated school-related problem.

Family, peer, and school-related factors may also influence bullying involvement. Difficulties establishing or maintaining friendships, economic difficulties, reduced school connectedness, and frequent communication between schools and families because of behavioral problems were associated with victimization or perpetration [27]. Lower social preference and poorer teacher–student relationships were also associated with greater bullying involvement [33], while lower family satisfaction was related to more severe victimization and perpetration among adolescents with ADHD [23]. This finding is consistent with previous evidence suggesting that positive teacher–student relationships and low levels of teacher–student conflict may reduce bullying involvement by fostering more supportive classroom environments [38]. Conversely, positive school climate and greater parental monitoring were associated with a lower likelihood of traditional and cyberbullying victimization [35]. These findings support a broader understanding of bullying in which the characteristics of the child and the environments surrounding them may contribute to increasing or reducing vulnerability to different forms of bullying involvement.

The available evidence also highlights the heterogeneity of bullying experiences among children and adolescents with ADHD. One study reported greater involvement among males in most forms of bullying [29], whereas emerging evidence suggests that ADHD symptoms among adolescent females may be associated with multiple forms of victimization and greater involvement in perpetration; among males, this association appeared more limited and was observed mainly for verbal abuse [36]. A particularly high burden of anxiety and depressive symptoms associated with victimization was also observed among females [31]. Similarly, psychiatric and neurodevelopmental comorbidities may increase vulnerability to bullying involvement. Comorbid ASD was associated with greater victimization [23,24], while the coexistence of ADHD with other psychiatric disorders was associated with greater bullying involvement, particularly bully–victim experiences [32]. Differences according to ADHD presentation may also be relevant, as greater victimization was observed among preadolescents with the combined presentation of ADHD [24]. Although the available evidence remains limited, these findings suggest that bullying involvement may vary according to individual and clinical characteristics and reinforce the need to avoid considering young people with ADHD as a homogeneous population.

The emergence of cyberbullying further extends the contexts in which bullying involvement may occur. Cyberbullying has become an increasingly relevant concern among children and adolescents worldwide, with evidence indicating a significant increase in its prevalence over time [39]. An ADHD diagnosis during childhood was associated with an increased risk of both traditional bullying victimization and cyberbullying victimization during adolescence [35]. This finding suggests that vulnerability to victimization may persist beyond face-to-face interactions. Digital environments therefore represent an additional context that should be considered when examining bullying experiences in this population.

Taken together, the available evidence supports an integrative perspective in which bullying involvement among children and adolescents with ADHD cannot be explained by ADHD symptoms in isolation. Instead, individual characteristics, peer experiences, emotional difficulties, and family and school contexts appear to contribute to different patterns of involvement. The reciprocal relationship between inattentive symptoms and victimization [34], together with the coexistence of victim and perpetrator roles [23,27,28,30,32,36], further suggests that these experiences may change and interact over time. Although the current evidence does not allow causal pathways to be established, bullying involvement in young people with ADHD may be understood as a multifactorial and developmentally interconnected phenomenon.

### 4.1. Future Research Directions

Future studies should further investigate the role of comorbidities, particularly comorbidity with ASD, to better understand how these conditions may modify the risk of victimization and perpetration among children and adolescents with ADHD, as well as whether bullying involvement differs according to ADHD presentation (combined, predominantly inattentive, and predominantly hyperactive-impulsive), given the currently limited evidence. Likewise, future research should examine sex differences in greater detail, as the available evidence suggests distinct patterns of bullying involvement between males and females, although the mechanisms underlying these differences remain unclear. Evidence from general adolescent populations also suggests that bullying involvement and the factors associated with intimidation may differ between males and females, further supporting the need for sex-specific analyses in future research [40]. Such analyses should also consider the potential impact of the underrecognition and underdiagnosis of ADHD in females when interpreting sex-related differences. Future research should also consider the potential influence of socioeconomic and cultural contexts on bullying involvement among children and adolescents with ADHD.

In addition, longitudinal and interventional studies are needed to evaluate the potential protective effects of different factors. Further research should examine the role of teacher–student relationships, school climate, and peer relationships, as the available evidence suggests that these factors may influence vulnerability to bullying. Finally, considering the reported benefits of physical activity and exercise on executive functions, behavioral regulation, and quality of life in children and adolescents with ADHD [41,42,43], together with emerging evidence linking physical activity, emotional management, interpersonal relationship distress, and bullying involvement in school populations [44], future studies should examine whether school-based physical activity and exercise programs could serve as a complementary strategy to promote social integration and reduce involvement in bullying among young people with ADHD.

### 4.2. Practical Implications

The findings of the present review have important implications for both clinical practice and educational settings. Prevention and intervention strategies for bullying among children and adolescents with ADHD should consider not only ADHD-related characteristics but also the family, school, and social factors associated with bullying involvement. Consequently, comprehensive approaches that address both individual difficulties and contextual vulnerabilities may be particularly relevant [45].

Furthermore, considering that children and adolescents with ADHD show greater involvement as victims, perpetrators, and bully–victims, as well as an increased risk of cyberbullying, assessments in both clinical and school settings should consider all these forms of involvement. Likewise, the observed differences between males and females suggest that prevention and intervention strategies should be adapted according to the characteristics of each group.

Finally, the findings highlight the potential protective role of families, teachers, peer relationships, and a positive school climate. In this context, schools should promote multidisciplinary interventions aimed at strengthening these protective factors, fostering social inclusion, and facilitating the early identification of students at greater risk of becoming involved in bullying. This approach is consistent with broader evidence supporting comprehensive multilevel approaches and school-based strategies to address bullying among children and adolescents [45,46].

## 5. Conclusions

The available evidence indicates that children and adolescents with ADHD show greater involvement in different forms of bullying, including victimization, perpetration, bully–victim profiles, and cyberbullying. However, the findings of this review suggest that this association is not explained solely by the core symptoms of the disorder but rather reflects the interaction between individual, emotional, family, school, and social factors.

Taken together, the available evidence highlights the importance of understanding bullying among young people with ADHD from a multifactorial perspective, considering the role of peer relationships, family, teachers, and the school environment, as well as the observed sex differences and the influence of specific comorbidities. This approach provides a broader understanding of the phenomenon and offers a foundation for the development of more comprehensive prevention strategies and interventions.

Finally, strengthening protective factors across the different developmental contexts of children, promoting early identification, and designing multidisciplinary interventions tailored to the needs of this population may contribute to reducing bullying involvement and promoting the well-being of children and adolescents with ADHD.

## Data Availability

No new data were created or analyzed in this study. Data sharing is not applicable to this article.

## Supplementary Materials

The following supporting information can be downloaded at: https://www.mdpi.com/article/10.3390/children13081047/s1, The PRISMA Checklist.

## Abbreviations

The following abbreviations are used in this manuscript:

ADHD	Attention-deficit/hyperactivity disorder
ASD	Autism spectrum disorder
ADHD-C	Attention Deficit Hyperactivity Disorder-Combined type
OR	Odds ratio
